# Does N200 Reflect Semantic Processing?—An ERP Study on Chinese Visual Word Recognition

**DOI:** 10.1371/journal.pone.0090794

**Published:** 2014-03-12

**Authors:** Yingchun Du, Qin Zhang, John X. Zhang

**Affiliations:** 1 Key Laboratory of Behavioral Science, Institute of Psychology, Chinese Academy of Sciences, Beijing, China; 2 Department of Psychology, Capital Normal University, Beijing, China; 3 Department of Psychology, Fudan University, Shanghai, China; Stony Brook University, United States of America

## Abstract

Recent event-related potential research has reported a N200 response or a negative deflection peaking around 200 ms following the visual presentation of two-character Chinese words. This N200 shows amplitude enhancement upon immediate repetition and there has been preliminary evidence that it reflects orthographic processing but not semantic processing. The present study tested whether this N200 is indeed unrelated to semantic processing with more sensitive measures, including the use of two tasks engaging semantic processing either implicitly or explicitly and the adoption of a within-trial priming paradigm. In Exp. 1, participants viewed repeated, semantically related and unrelated prime-target word pairs as they performed a lexical decision task judging whether or not each target was a real word. In Exp. 2, participants viewed high-related, low-related and unrelated word pairs as they performed a semantic task judging whether each word pair was related in meaning. In both tasks, semantic priming was found from both the behavioral data and the N400 ERP responses. Critically, while repetition priming elicited a clear and large enhancement on the N200 response, semantic priming did not show any modulation effect on the same response. The results indicate that the N200 repetition enhancement effect cannot be explained with semantic priming and that this specific N200 response is unlikely to reflect semantic processing.

## Introduction

Reading is a highly emphasized and much cultivated core cognitive skill in modern society. How we recognize words has been the focus of a myriad of investigations employing a range of different methodologies. Most previous research has focused on the processing of simple words, and interest on the recognition of complex words has been emerging only recently [1]. Complex words are particularly important for Chinese as its vocabulary is dominated by compound words which are morphologically complex words formed by two or more constituent morphemes. Each morpheme is typically represented by a single character which is not necessarily a simple word. At an intermediate level between characters, simple words and sentences, compound words reflect the properties of both lexical representations and grammatical processing, therefore offer a unique opportunity to understand the interplay between storage and computation in the mind [2].

Repetition priming and semantic priming are key tools in the study of a wide range of word-related processes [3]. Repetition priming refers to the facilitated processing of a stimulus on repeated presentation relative to its initial presentation [4]. Brain correlates of repetition priming have been explored in hemodynamic studies [5]–[8] and in electrophysiological studies [9]–[14]. It is well-known that repeated stimulus presentations elicit a relatively reduced neural activation, referred to as “repetition suppression”. Earlier studies in the literature revealed two types of word repetition effects: (1) an initial modulation of the waveform in the region of P200, with ERPs to repeated words showing a transient negative-going deflection (less positive-going P200); (2) a sustained positive-going shift with an onset of approximately 300 ms and with increased amplitude for repeated words [15], [16]. By investigating the repetition effect in relatively natural discourse structure instead of in experimental lists of words, Van Petten et al. found three distinct ERP components sensitive to repetition: an early P2 enhancement (more positive), a subsequent reduction in N400, and a substantial amplitude reduction in a late positive component (LPC) [17]. Similar P2 repetition effect was found in a study by Evans and Federmeier who linked the effect to implicit recognition processes [18]. ERPs elicited by repeated words are generally more positive-going compared with new words [19]–[21], although the repetition effects vary across different studies regarding their onset, amplitude modulation, and duration [22].

Semantic priming is the facilitated processing of a target word when it is preceded by a semantically related prime word, compared with when it is preceded by an unrelated prime word [23], [24]. Word repetition effects have been shown to be independent of semantic priming effect [25]. Semantic priming effects are thought to be entirely due to semantic relationships or associative links between primes and targets [24], whereas several additional types of information (e.g., orthographic, phonological) are presumed to contribute to repetition priming effects [15], [26]. Typically, repetition priming effects are substantially greater and longer lasting than semantic priming effect [8], [26], consistent with the view that repetition and semantic priming do not arise at least entirely from the operation of a common mechanism. Besson et al. [27] indicated that the N400 is sensitive to both semantic congruity and repetition. Despite N400, there are studies suggesting that lexical semantic access might occur earlier, within 200 of the stimulus presentation [28]. Sereno et al. [29] found that the amplitude of N1 component occurring between 132 and 192 ms after stimulus onset was sensitive to semantic context.

Combined repetition priming and semantic priming with lexical decision task, Zhang et al. conducted a series of ERP experiments on the recognition of two-character Chinese compound words [30]. During the experiment, participants were presented with a series of 2-character real words and pseudowords and asked to discriminate between the two. Different experimental conditions were defined based on the relationship between 2 neighboring stimuli (the n and n+1 stimulus) to include 6 types of prime-target word pairs: (1) control condition, the two were unrelated real words (*钱币-微弱*, money-weak); (2) phonological priming condition, the two were homophones (same tone as well) but different in orthography and unrelated in semantic meaning (*电源-店员*, power-clerk); (3) semantic priming condition, the two were semantically related but different in orthography and phonology (*白云-蓝天*, white cloud - blue sky); (4) whole-word repetition condition, the two were the same real word (*思索-思索*, think-think); (5) first-character repetition condition, the two compound words shared the first character but not the second (*荣幸-荣华*, honored-prosperity); (6) second-character repetition condition, the two compound words shared the second character but not the first (*流利-互利*, fluent-benefiting).

Compared with the control condition, they found N400 attenuation for the semantic priming and repetition priming conditions, consistent with typical findings in the literature [31], [32]. Critically, they observed an ERP response peaking about 200 ms following the onset of a compound word. Considering its broad distribution in central and parietal regions, the response was tentatively referred to as the centro-parietal N200. A special feature of this N200 is that its magnitude would be significantly enhanced at a word's second presentation (i.e., larger in condition 4 compared with condition 1), opposite to N400's reduction under repetition priming. The N200 enhancement effect was also present for condition 5 and 6, though smaller in effect size compared with condition 4, consistent with the fact that prime-target words were only partially similar in orthography. In addition, the N200 seems to be unaffected by phonological priming or semantic priming (i.e., no difference between condition 2 and condition 1, and between condition 3 and condition 1).Based on these findings, Zhang et al. interpreted the observed N200 as a brain response associated with orthographical processing in Chinese compound word recognition. Similar partial orthographic repetition effect on N200 was replicated in Jia et al. study [33].

As we are unaware of other published studies describing similar enhanced N200 priming effect as reported in Zhang et al. [30], more research is needed to characterize the nature of this response. As an important first step, more evidence is needed to test the notion that this N200 is associated with the orthographical but not the semantic aspect of lexical processing. Since semantic priming in Zhang et al. [30] was manipulated cross trials and the two items were separated by more than a few seconds (3.4∼3.6 s), semantic information may dissipate during these delay intervals and therefore fail to affect the N200.

In the present study, we conducted two experiments to examine whether or not this N200 is indeed unrelated to semantic processing. In the first experiment, we used a more classical within-trial priming paradigm. We intended to enhance semantic priming effects by reducing the prime-target interval in a within-trial priming design. If the N200 were not affected by such semantic manipulation, it would provide stronger evidence that it is not related to semantic processing.

Secondly, Zhang et al. [30] used lexical decision tasks that only engage semantic processing implicitly. Here in Experiment 2, a semantic relatedness judgment task was used to ensure meaning access for both the target and the prime items. Under such a task explicitly engaging semantic processing, if N200 were still unaffected by semantic priming, it would present further evidence that N200 does not reflect semantic processing.

## Experiment 1: Primed Lexical Decision Task

### Methods

#### Participants

Twenty native Chinese-speaking college students (11 male, age range from 19 to 26 years, mean ± SD = 22.5±1.8 years) participated in this experiment with monetary compensation. All were right-handed with normal or correct-to-normal vision. None of them reported any neurological or psychiatric diseases. This study was approved by the Institutional Review Board of Institute of Psychology, Chinese Academy of Sciences, Beijing, China. Written informed consent was obtained in accordance with guidelines from the IRB of Institute of Psychology.

#### Materials

There were three critical experimental conditions each with 84 prime-target pairs: 1) Repetition condition: the targets were identical to the primes; 2) Related condition: the primes and targets were semantically related but shared no constituent characters; 3) Control condition: the primes and targets were semantically unrelated and shared no constituent characters. Both primes and targets in the critical conditions were real two-character compound words. To eliminate the possibility that the ERP effect was caused by differences in target stimulus characteristics, each target was paired with all types of prime across participants. The semantic relatedness was rated by 10 college students who did not participate in the ERP experiment using a 5-point scale (1 for unrelated in meaning and 5 for highly related). The mean relatedness between the word pairs was 3.6 (SD = 0.39). Some examples of the stimuli are listed in Table 1.

**Table 1:** Stimulus examples for the experimental conditions in Experiment 1, with the first two-character word as the prime and the second word as the target.

Repetition	Semantic-related	Control	Filler
偶然	方法-途径	现在-差别	沙漠-独讯
occasional	method-approach	now-difference	desert-
积极	国王-皇帝	古老-分析	色彩-福怒
active	king-emperor	ancient-analysis	color-
灯光	奢侈-贵族	取暖-降低	挖掘-通然
lamplight	luxury-nobility	heating-lower	dig-
泡沫	注册-登记	融化-出版	尖端-传千
foam	register-enroll	melt-publication	cusp-
回答	绿色-蔬菜	飞翔-反对	枯燥-奉惕
answer	green-vegetable	fly-against	baldness-
锻炼	巴结-奉承	植物-认真	镇定-紧由
practice	flatter-compliment	plant-seriously	calm-
选拔	金钱-财产	漫长-玉米	机会-威溉
election	money-fortune	endless-corn	opportunity-
拖延	文明-礼貌	经历-密切	气息-奖阔
delay	civilization-courtesy	experience-closely	breath-
狐狸	从前-曾经	深刻-钢铁	坦白-预员-
fox	aforetime-once	profundity-iron	confess
扩张	待遇-福利	安静-论文	紧急-登力
dilation	treatment-welfare	quiet-thesis	emergency-

The target words in the filler trials were nonsense pseudowords.

A total of 252 prime-target pairs using pseudowords as the target constituted the filler trials. The pseudowords were created by concatenation of two characters that do not occur in the real word corpus. Also, the pseudowords were not homophonic to any real word. Characters used for pseudowords did not overlap with characters used for real words. The visual complexity (i.e., stroke number) was matched between the critical words and the pseudowords. Word frequency was measured according to an online corpus based on a research project of Middle Tennessee State University (http://lingua.mtsu.edu/chinese-computing/introduction.html). The mean stroke number of the real words (sum of the two characters) was16.7 (SD = 4.5), and the mean word frequency was 85 occurrence per million (SD = 143). The mean stroke number of pseudowords was 17.1 (SD = 4.7).

#### Procedure

Participants were seated in a dimly lit and sound-attenuated room. All visual stimuli were presented on a computer monitor that was about 1 m away from participants' eyes. All word stimuli were displayed at high contrast as black words on a white background, subtending a visual angle of 4.3°×2.3°. Participants were instructed to remain relaxed and to refrain from moving throughout the experiment.

After being familiarized with a practice block, each participant completed 6 test blocks. Both practice block and test block contain 72 trials. In one block, there were 12 trials for each critical condition and 36 trials for filler condition with pseudoword as the target. The different types of trials were randomly intermixed. Each trial began with a black cross presented centrally for 500 ms. The prime was then presented and remained on screen for 400 ms. The target was presented for 400 ms after a 1200 ms blank interval. The inter-trial-interval varied randomly from 1200 to1400 ms. Participants were instructed to attend to both words but respond only to the target by judging whether it was a real word or not. Response speed and accuracy were equally emphasized. The response and key mappings were counterbalanced across participants.

#### ERP recording and analysis

The electroencephalogram (EEG) was recorded from the scalp with 64 nonpolarizable Ag/AgCl sintered electrodes using a Neuroscan system with a sampling rate of 500 Hz. The electrode sites followed the extended 10–20 convention. All electrode impedance was maintained below 5 kΩ. In addition to the scalp sites, the horizontal EOG was recorded at the outer canthi of both eyes and the vertical EOG was recorded between supraorbit and suborbit of the left eye. Nose tip was used as the recording reference. Reference was changed offline to the average of the two mastoids.

Average ERPs were computed offline for correct trials free of ocular and movement artifacts. Eye movement artifacts were removed using regression-based weighting coefficients. EEG segments were abstracted from 100 ms before stimuli onset to 900 ms post stimuli onset. The 100 ms pre-stimuli period was used as the baseline. The segments were baseline corrected and band pass filtered (0.5–30 HZ). Segments with amplitude exceeding ±80 µv in any scalp channel were excluded from analysis (less than 2% of trials rejected). Averaged ERPs were computed separately for the target items in the three critical conditions. Filler items were not analyzed. Grand-average waveforms were derived from individual ERPs.

### Results and discussion

For all participants, response was highly accurate and fast. Table 2 shows the behavioral results for all conditions. The mean RTs were 605 ms (SD = 103), 650 ms (SD = 109), 676 ms (SD = 105), and 733 ms (SD = 136) for the Repetition, Related, Control and filler conditions, respectively. The corresponding error rates were 1.1% (SD = 1.8), 2.2% (SD = 2.4), 4.4% (SD = 4.5), and 4.2% (SD = 2.8). The results indicated that all participants followed the instructions and were attentive to the word pairs during the experiment. ANOVA on RT revealed a significant main effect of trial condition (F(3,57) = 53.1, *p*<0.0001). Post-hoc comparisons indicated that RT in the Repetition condition was significantly shorter than in both the Related and the Control conditions (605 vs. 650 ms, t(19) = 6.5, *p*<0.0001; 605 vs. 676 ms, t(19) = 9.6, *p*<0.0001), and RT in the Related condition was significantly shorter than in the Control condition (t(19) = 5.7, *p*<0.0001). For error rates, there was a significant main effect of trial condition (F(3,57) = 7.6, *p*<0.001), with the Control trials being less accurate than the Repetition trials (4.4% vs. 1.1%, t(19) = 4.0, *p*<0.001) and the Related trials (4.4% vs. 2.2%, t(19) = 2.8, *p*<0.01). Briefly, the RT results showed significant repetition priming and semantic priming effects, with larger effect size for the former (71 ms) than for the latter (45 ms). The error rates showed a consistent pattern.

**Table 2:** Mean response times and error rates for the four experimental conditions (N = 20).

	*Repetition*	*Semantic-Related*	*Control*	*Filler*
RT (ms)	605±103	650±109	676±105	733±136
Error Rate (%)	1.1±1.8	2.2±2.4	4.4±4.5	4.2±2.8

**Figure 1:**
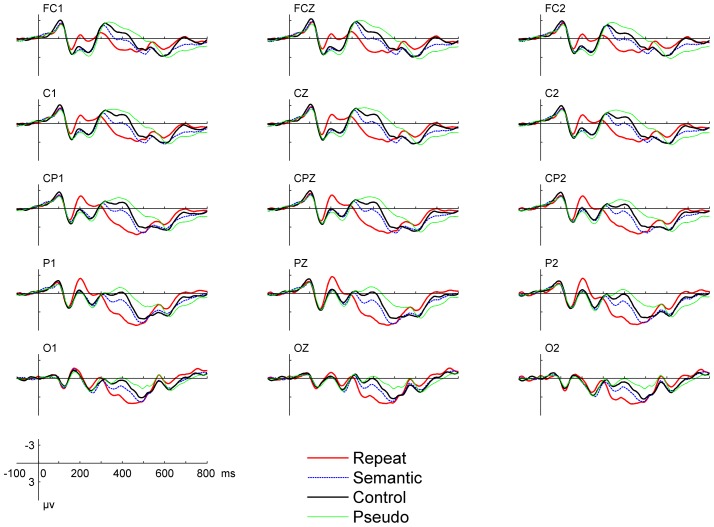
ERP waveforms for all experimental conditions in Exp. 1. Repeated stimuli induced larger negative shift in the N200 time window.

**Figure 2:**
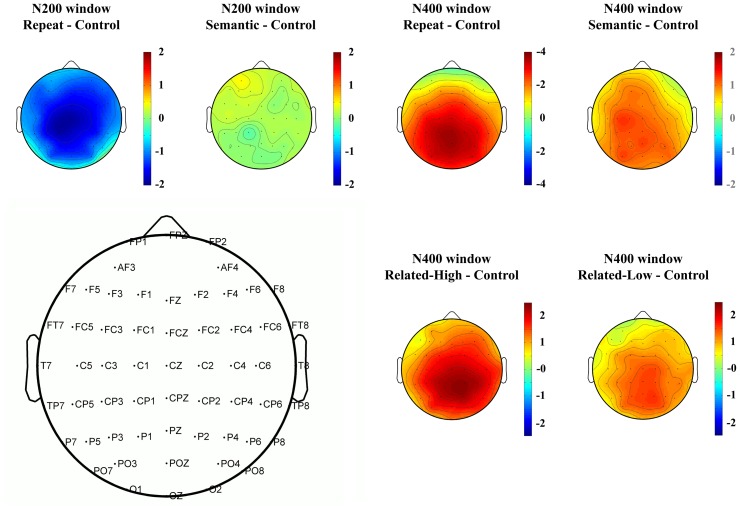
Scalp distribution for difference waves. The upper panel shows topographical distribution in N200 and N400 time windows for Exp. 1, and the lower panel shows topographical distribution in N400 time window for Exp. 2. The N200 time window results are not plotted for Exp. 2 due to absence of effects. Note scale differences in different maps.

The grand-average waveforms for all conditions are plotted in Figure 1 for representative electrodes. Salient ERP response differences across different conditions were found for both the N200 and the N400 components. To help visualize the distribution of the repetition effect reflected by N200 and N400, topographical voltage maps based on difference waves were illustrated in the upper panel of Figure 2. Repeated-measures ANOVAs with Geisser–Greenhouse correction were performed on the averaged amplitude of N200 and N400, with trial type, laterality (left hemisphere, middle, and right hemisphere), and electrode position (fronto-central: FCZ, FC1/2; central: CZ, C1/2; centro-parietal: CPZ, CP1/2; parietal: PZ, P1/2) as factors.

The mean amplitude of N200 was measured from the 160–220 ms time window. The main effects were significant for trial type, F(2,38) = 29.7, *p*<0.0001, and for electrode position, F(3,57) = 12.0, *p*<0.0001. No interaction was significant. Follow-up comparisons showed significantly larger negative shifts of N200 in the Repetition condition than in the Related condition (−0.5 vs 1.2 µv, F(1,19) = 36.8, *p*<0.0001) and in the Control condition (−0.5 vs 1.1 µv, F(1,19) = 46.3, *p*<0.0001). The Related condition was not different from the Control condition (1.2 vs. 1.1 µv, *p*>0.5). Clearly, N200 was enhanced in a very prominent way in the repetition priming condition but not changed at all in the semantic priming condition.

The averaged amplitude of N400 was measured from the 360–460 ms time window. The main effect was significant for trial type, F(2,38) = 43.8, *p*<0.0001, and for electrode position, F(3,57) = 31.4, *p*<0.0001. Follow-up comparisons showed smaller amplitude (more positive) for the Repetition condition than the Related (2.9 vs. 0.8 µv, F(1,19) = 43.2, *p*<0.0001) and the Control conditions (2.9 vs. −0.3 µv, F(1,19) = 54.7, *p*<0.0001). The amplitude for the Related condition was also significantly lower than the Control condition (0.8 vs. −0.3 µv, F(1,19) = 16.5, *p*<0.001). As typically found in the literature, N400 showed a highly significant amplitude reduction for repetition priming and a smaller but still significant reduction for semantic priming. The interaction between trial type and laterality was also significant, F(4,76) = 2.8, *p*<0.05. For the Repetition and Related conditions, laterality effects were not significant (*ps*>0.1). The mean amplitudes were 2.8 µv for the left hemisphere, 3.0 µv for the midline, 3.0 µv for the right hemisphere under the Repetition condition. For the Related conditions, the values were 0.8 µv for the left hemisphere, 0.7 µv for the midline, 0.9 µv for the right hemisphere. For the Control condition, laterality effect was marginally significant, F(2,38) = 3.2, *p* = 0.053. The mean amplitudes were −0.3 µv for the left hemisphere, −0.4 µv for the midline, −0.1 µv for the right hemisphere.

## Experiment 2: Semantic Relatedness Judgment

### Methods

#### Participants

Twenty right-handed, native Chinese-speaking college students (9 male, age range from 19 to 24 years, mean ± SD = 22.1±1.5 years) participated in this experiment. Other information was the same as in Exp. 1.

#### Materials

The stimuli were formed from 264 pairs of compound words. Each pair of words were related in meaning either at a high level (Related-High, e.g., *玻璃-透明*, Glass - Transparent) or a low level (Related-Low, e.g., *玻璃-水晶*, Glass - Crystal), or unrelated at all (Control, e.g., *石头-天空*, Sky - Stone). More examples are listed in Table 3. Using a 5-point scale (1 for unrelated in meaning and 5 for highly related), the relatedness of each word pair was rated by the same 10 college students rated in Exp. 1. The relatedness between word pairs was 3.5 (SD = 0.39) for the Related-High pairs, 3.0 (SD = 0.38) for the Related-Low pairs, and 1.24 (SD = 0.36) for the Control pairs. Significant difference was found in semantic relatedness in all three pair-wise comparisons (*ps*<0.0001). The mean stroke numbers of prime words (sum of the two characters) were 16.1 (SD = 4.8) for the Related-High condition, 16.5 (SD = 4.1) for the Related-Low condition, and 16.2 (SD = 4.5) for the Control condition. The word frequencies were 86 (SD = 160) occurrence per million for Related-High primes, 85 (SD = 139) for Related-Low primes, and 98 (SD = 195) for Control primes. The mean stroke numbers were 17.3 (SD = 4.9) for Related-High targets, 18.0 (SD = 5.0) for Related-Low targets, and 17.6 (SD = 5.3) for Control targets. The frequencies were 94 (SD = 195) occurrence per million for Related-High targets, 78 (SD = 165) for Related-Low targets, and 91 (SD = 131) for Control targets. For both the prime and the target, frequency and stroke numbers were matched across the three conditions (*p*>0.3).

**Table 3:** Stimulus examples for the experimental conditions in Experiment 2, with the first two-character word as the prime and the second word as the target.

Related-high	Related-low	Control
强大-力量	导线-金属	情感-骆驼
powerful-strength	wire-metal	feeling-camel
立刻-紧急	工作-生活	号码-自由
immediate-emergency	work-life	number-freedom
魅力-吸引	毅力-勤奋	郁闷-迅速
charm-attraction	willpower-diligence	depression-fast
白天-太阳	影响-榜样	抵抗-现在
daylight-sun	influence-model	resist-now
负担-沉重	珍贵-友谊	合格-冰川
burden-heavy	precious-friendship	qualified-iceberg
卫生-干净	激动-情绪	弘扬-多疑
sanitation-clean	excite-emotion	propagate-suspicious
报复-仇恨	放弃-失败	资金-忽然
revenge-hatred	abandon-failure	bankroll-sudden
石像-雕塑	性格-活泼	理性-军队
statue-sculpture	disposition-breeziness	rational-army
挫折-打击	敏感-反应	利息-反对
frustration-shock	sensitive-reaction	accrual-against
车辆-交通	荒地-孤立	跑步-官僚
vehicle-traffic	wasteland-isolation	run-bureaucracy

All items were real words.

After 24 practice trials, participants completed 4 test blocks, each containing 15 Related-High trials, 15 Related-Low trials, and 30 Control trials. The different types of trials were randomly intermixed. Each trial began with a cross fixation, followed by a 500 ms prime word. After an inter-stimulus-interval varying randomly from 400–600 ms, the target word was presented for 500 ms, and then replaced by a dot with 1300 ms duration. Participants were instructed to attend to both words and to judge whether or not the two were semantically related. Response made from the target onset till the dot disappearance was accepted as valid response. All other aspects of the experiment, including procedure, data recording and analysis, were the same as in Experiment 1.

### Results and discussion

Data from one participant was excluded from analysis because of high error rate. Table 4 shows the behavioral results for all conditions. The mean RTs were 783 ms (SD = 133), 836 ms (SD = 146), and 859 ms (SD = 158) for the Related-High, Related-Low, and Control conditions, respectively. The corresponding error rates were 6.3% (SD = 6.0), 12.4% (SD = 8.4), and 6.2% (SD = 4.7). ANOVA on RT revealed a significant main effect of trial condition, F(2,36) = 17.9, *p*<0.0001. Post-hoc comparisons indicate that RTs of the Relate-Low condition and the Control condition were slower than that of the Related-High condition (836 vs.783 ms, t(18) = 7.1, *p*<0.0001; 859 vs. 783 ms, t(18) = 4.5, *p*<0.001). RT of the Related-Low condition was faster than the Control condition, though not significant (836 vs. 859 ms, t(18) = −1.74, *p*<0.1). For error rates, there was a significant main effect of trial condition, F(2,36) = 6.2, *p*<0.005, with the Related-Low condition being less accurate than the Related-High condition (12.4% vs. 6.3%, t(18) = 4.7, *p*<0.001) and the Control condition (12.4% vs. 6.2%, t(18) = 2.4, *p*<0.01).

**Table 4:** Mean response times and error rates for the three experimental conditions (N = 19).

	*Related-High*	*Related-Low*	*Control*
RT (ms)	783±133	836±146	859±158
Error Rate (%)	6.3±6.0	12.4±8.4	6.2±4.7

The faster RT in the Related-High condition compared with the Control condition demonstrates a significant semantic priming effect of 76 ms. Across the two conditions, the error rates were comparable. The semantic priming effect was 23 ms in the Related-Low condition, much smaller in effect size and not reaching significance. This indicates that the semantic association between the prime and target in the Related-Low condition was subjectively not very strong to the participants. This is also evidenced by the high error rate in this condition reflecting participants' uncertainty in judging the semantic relatedness between two weakly-related items.

**Figure 3:**
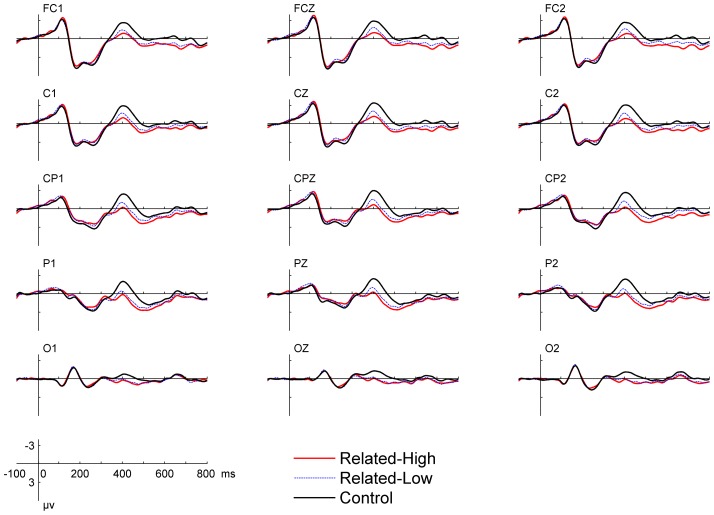
ERP waveforms for all experimental conditions in Exp. 2. There is no experimental effect in the N200 time window.

Grand-average waveforms for all experimental conditions are plotted in Figure 3. The waveforms showed a plateau in the 160–220 ms time window. Even without a peak, this plateau already signifies the occurrence of N200. This is because the negative-going N200 lies within the much larger positive-going P200 component and the presence of N200 can be inferred from its modulation of the P200 valley by making it less deep and sharp. By visual inspection, the N400 was clearly different across conditions. The lower panel of Figure 2 shows the topographical map based on the mean amplitudes of difference waves measured within the N400 time window.

Statistical analysis was performed on the N200 and N400 components with averaged amplitudes measured in the 160–220 ms time window for N200 and in the 360–460 ms time window for N400. Repeated-measures ANOVAs were conducted on the averaged amplitude of N200 and N400, with trial type, laterality (midline, left hemisphere and right hemisphere), and electrode position (fronto-central: FCZ, FC1/2; central: CZ, C1/2; centro-parietal: CPZ, CP1/2; parietal: PZ, P1/2) as factors. Within the N200 time window, no significant difference was found across different trial types, F(2,36) = 0.7, *p*>0.1. The mean amplitudes were 2.3 µv, 2.3 µv, and 2.7 µv for the Related-High, Related-Low, and Control conditions, respectively. The null result suggested that the N200 was not modulated by the semantic relatedness between the word pairs. For the N400 component, there was a main effect for trial type, F(2,36) = 17.4, *p*<0.0001, for laterality, F(2,36) = 8.4, *p*<0.01, and for electrode position, F(3,54) = 8.5, *p*<0.0001. The interaction between electrode position and laterality was also significant, F(6,108) = 2.2, *p*<0.05.

Follow-up comparisons showed significant amplitude reduction for the Relate-High condition than the Control condition (−0.1 vs. −2.1 µv, F(1,18) = 27.2, *p*<0.0001), and for the Related-Low condition than the Control condition (−0.6 vs. −2.1 µv, F(1,18) = 33.4, *p*<0.0001), demonstrating clear effects of semantic priming. The N400 amplitude tended to be reduced from the Related-Low condition to the Related-High condition, though not significant (−0.6 vs. −0.1 µv, F(1,18) = 2.0, *p* = 0.17).

By the RT measure, the semantic priming effect in the Related-low condition was not significant. The error rate of the Related-low condition was even higher than that of the Control condition. Possibly the semantic association between the prime and target in the Related-Low condition was not strong enough to show semantic priming in the behavioral measures. However, semantic priming was revealed in N400, the amplitude of which was significantly smaller in the Low-related condition than the Control condition, likely due to the greater sensitivity of the ERP measures.

## General Discussion

The present study used two tasks engaging semantic processing either implicitly or explicitly to examine whether the N200 response found in Zhang et al. [30] is modulated by semantic manipulations.

In Exp. 1 with the lexical decision task, participants judged whether or not a target word was a real word. Response time to the target was significantly faster when it was a repetition of the prime or semantically related to the prime, demonstrating clear repetition priming and semantic priming behaviorally. The effect size was smaller for the semantic priming than for the repetition priming. This is a sensible result as semantic priming involves only partial re-presentation of semantic information, while repetition involves re-presentation of orthography, phonology, and semantic information in full.

Replicating our previous findings, repetition priming produced a clear and large enhancement on the N200 amplitude [30], [33]. The effect size measured in terms of the mean amplitude increment from the control condition was 1.6 µv, larger than that in the corresponding conditions in all four experiments in our previous study (Exp. 3–6: 0.7, 1.0, 0.8, 1.4 µv). We attribute this large effect size to the change from the previous across-trial design to the more sensitive with-trial design reducing the temporal lag between the prime and the target. This replication of the repetition enhancement effect confirms the central characteristic of this N200 response that distinguishes it from other negative ERP responses in the same time window. Opposite to N200, N400 showed a clear amplitude reduction for repetition priming, consistent with literature findings and with our previous experiments [30]. This indicates that the second time a word is perceived, its semantic processing is facilitated.

What is most critical is that the amplitude of the N200 did not change regardless of whether or not the prime and the target were semantically related. As semantic priming was found from both the behavioral data and the N400 response, this negative finding cannot be attributed to lack of power but indicates clearly that the N200 response is unlikely to be sensitive to semantic processing.

In Exp. 2 with the semantic relatedness judgment task, participants explicitly determined whether or not the prime and target were related in meaning. When the two items were strongly related semantically, the behavioral results showed clear semantic priming. When they were more weakly related, the results showed the same trend although not reaching significance. Even so, N400 reduction was significant for both strongly and weakly related items, suggesting that the semantic overlap between the prime and target were neurally registered and facilitated the processing of the target.

Critically, the N200 was not modulated by the semantic relatedness between the prime and the target, showing no difference across the Related-High, Related-Low, and the Control conditions. Again, given the evidence for semantic priming from both the behavioral data and the N400 response, this negative result cannot be attributed to lack of power, i.e., the semantic manipulation was not strong enough.

Combining the two experiments, the results show that while N200 was strongly affected by repetition (identity) priming, it was not sensitive to semantic priming. As repetition priming with linguistic materials can be generated from processing along three dimensions of orthography, phonology, and semantics, this finding indicates that the N200 found in Zhang et al. [30] is likely associated with either orthographical or phonological processing but not semantic processing. This is consistent with our previous proposal that this N200 reflects orthographical processing, given there is other evidence that this N200 is not sensitive to phonological priming, either.

Briefly, using a more sensitive semantic priming paradigm (Exp. 1) and a task explicitly engaging semantic processing (Exp. 2), the present study failed to find any evidence that the N200 enhancement effect is sensitive to semantic manipulations. The results strengthen the conclusion in Zhang et al. that this N200 response does not reflect semantic processing [30].

More research is needed to address the nature of this N200 response and particularly the nature of its enhancement upon repetition. Compared with N400, the functional significance of this N200 is still unclear. The traditional N200, also called N2 in some literature, refers to the second negative wave peaking between 200 and 350 ms after stimulus onset. It is suggested that the N200 elicited by visual stimuli should be divided into at least three subcomponents: a fronto-central (anterior) component related to the detection of novelty or mismatch from a perceptual template when the eliciting stimuli are attended, a second fronto-central component related to cognitive control (encompassing response inhibition, response conflict, and error monitoring), and one or two posterior N2s related to some aspects of visual attention [34].

Other than the traditional N2s, similar electrophysiological responses to the present N200 response include the auditory mismatch negativity (MMN) [35] and the N250 component extensively investigated by Holcomb, Grainger and colleagues using a masked priming paradigm [36], [37]. The MMN has similar time course and frontal scalp distribution as the present N200, but repetition priming results in MMN amplitude reduction instead of enhancement. The difference between N250 and the present N200 lies in that N250 is typically demonstrated in masked priming paradigm with simple words and tends to have a parietal and occipital distribution while the present N200 is demonstrated with compound words in tasks not involving masking. More importantly, repetition priming results in N250 amplitude reduction instead of amplitude enhancement.

Except the above mentioned relative earlier components. There are several negative responses occurring in the 200–500 ms time windows, including the N300, N350, and N390 that may possibly be related to the N200 response studied here. Compared with the centrally-distributed N400 for general semantic processing, the N300 is more frontally distributed and typically elicited only by picture stimuli. It is considered to index rapid matching of visual input to stored semantic knowledge [38], [39]. This close tie with semantic processing makes it difficult to relate the N300 to the N200 we observed which is not sensitive to semantic manipulation. So is the case for the N390 response which is thought to index conceptual knowledge [40]. The N350 is a fronto-central negativity peaking between 200 and 500 ms [41], larger for pseudo and scrambled objects than intact known objects and familiar shapes [34], [41], [42] and showing inverted polarity at occipito-temporal sites [43]. It is proposed to be a neurophysiological marker for object model selection, reflecting the search for a stored structural description that matches the perceived image [41]. In this sense, it looks related to our N200 associated with identification of the orthographical representation of Chinese characters. However, Schendan and Kutas predicted smaller N350 for matched objects than non-matched ones and indeed N350 was larger for new than repeated objects [38], [44]. This is opposite to the N200 we found which is larger for repeated words than controls, and larger for normal Chinese characters than their mirror images [45].

To sum up, a major difference between the present N200 response and the usually reported repetition effect is that the present N200 showed repetition enhancement instead of repetition suppression. Repetition suppression is generally considered an effect of stimulus repetition *per se*, occurring independent of other psychological or neurophysiological variables. In contrast, cognitive variables including stimulus recognition, learning and explicit memory can bias repetition effects in BOLD response toward enhancement instead of suppression [46]. Studies using the oddball paradigm demonstrated that low-probability stimuli elicited a larger N2 and as the probability decreased, the N2 elicited by these events increased in amplitude [47], [48].

In the present study and in Zhang et al. [30], the repeated condition is less frequent than the non-repeated conditions. The enhanced N200 response elicited by the repeated words may possibly be caused by their low probability. This is an issue needing further research. Xu [49] duplicated the design of a word recognition study by Alvarez et al. [50] where repetition trials accounted for 40% of the total number of trials, excepting changing the stimuli to Chinese words and participants to Chinese readers. Xu observed a clear N200 enhancement effect that was completely absent in Alvarez et al., suggesting that the N200 response is unlikely to be attributed to probability factor only (see Zhang et al. [30] for a re-plot of the two contrasting results). Further, Jia et al. [33] showed that the N200 enhancement was still present even when the probability of the partial repetition trials (prime-target pairs sharing the first character) was two times than that of the non-repeated trials.

Besides, it is noteworthy that there may be a confounding factor between the repetition and the assigned task of lexical decision. Since only the real words were repeated and the pseudowords were not, detecting a repetition would signal the correct response of “word” in the lexical decision task. This design feature raises the possibility that the N200 effect is driven by the specific task configuration rather than some fundamental psycholinguistic processes. For example, in oddball paradigms with two sets of stimuli (only one of which calls for a response), a posterior N2 is larger for rare targets than for common nontargets and precedes a parietally maximal P3 [42]. Although the scalp distribution of the posterior N2 target effect is different from the present N200 effect, whether the confounding factor affects the present N200 effect should be further investigated with an experimental design including pseudoword repetition.

Kuperberg et al. used the same tasks to examine how task and semantic relationship modulate hemodynamic activity during lexico-semantic processing [51]. The lexical decision task was primarily associated with inferior prefrontal and ventral inferior temporal/fusiform hemodynamic response suppression to related word pairs. In contrast, the explicit semantic relatedness judgment task was primarily associated with left inferior parietal hemodynamic response enhancement to related word pairs. The authors explained the response suppression in the lexical decision task with pre-lexical automatic spreading activation and controlled expectancy, and response enhancement in relatedness judgment task with post-lexical semantic matching processes. According to literature findings, both anterior fusiform cortex [52] and left inferior prefrontal cortex [53], [54] contribute to the scalp-recorded N400, with the left temporal lobe as the largest source of the N400 semantic context effect [55].

These results are in accordance with the findings in the present study. Although semantic relationship leads to decrease of N400 amplitude in both tasks, there are differences in the pattern and peak latency of N400 across the tasks. The waveform of N400 in the lexical decision task showed two wave crests with peak latency around 300 ms, but only one crest with peak latency around 400 ms in the relatedness judgment task. As for the repetition condition in the lexical decision task, there was no need to use semantic expectancy strategy and the automatic spreading activation would have finished during prime presentation, resulting in a much reduced N400 with only one crest. The present results echo the viewpoint that the N400 can be decomposed into several functionally distinct subcomponents [56], although it should be noted that this experiment was not designed to test this notion.

Language difference may be the true reason for the absence of the N200 found by Zhang et al. [30] in the literature. In Chinese, most words are compound words constructed from a large set of visually complex block characters [57]. It has been suggested that written Chinese word recognition relies more on visual processing such as extraction of 2-dimensional form information and such processing underlies the N200 response examined here [30].

## Conclusions

Using both a primed lexical decision task and a semantic relatedness judgment task, the present study showed that a N200 response previously observed in Chinese compound word recognition is unaffected by semantic manipulations. Combined with previous results, the results suggest that the repetition enhancement effect characterizing this response is not caused by semantic priming. That this N200 response does not reflect semantic processing provides valuable insight to further understanding the nature of this seemingly novel ERP phenomenon.
